# Peritoneal Dialysis

**DOI:** 10.4061/2011/218974

**Published:** 2012-04-05

**Authors:** Hulya Taskapan, Olof Heimburger, Cengiz Utas, Paul Tam

**Affiliations:** ^1^Department of Nephrology, Inonu University, 44280 Malatya, Turkey; ^2^Karolinska Institutet, 171 77 Stockholm, Sweden; ^3^Erciyes University, 38039 Kayseri, Turkey; ^4^Scarborough General Hospital, ON, Canada M1P 2V5

Since the introduction of continuous ambulatory peritoneal dialysis by Moncrief and Popovich in 1976, the number of patients treated with peritoneal dialysis has continuously increased. Today, approximately 200 000 patients are treated with peritoneal dialysis (PD), and the growth is particularly rapid in many Asian countries.

Compared to hemodialysis, PD has been reported to be associated with similar patient survival (or even better during the first months) as well as better quality of life. Therefore, the PD first concept has been adopted in Hong Kong and Thailand. In this special issue about PD, K. Chaudhary et al. discuss the evidence for use of this concept. Then, the benefits and problems with use of PD in the rapidly growing diabetic population are discussed by A. Rocha and coworkers. Quality of life in PD patients is reported by M. Moreiras-Plaza et al., whereas P. Theofilou discusses socioeconomic factors and psychological problems such as depression and anxiety in patients with CKD.

Three further articles discuss different complications in PD patients: peritonitis (by M. OtsRosenberg et al.), calcific uremic arteriolopathy (by N. New and coworkers), and C. Kennedy et al. report a case series of patients with pleuroperitoneal leak, a rare but troublesome complication. 

Other four papers deal with the function of the peritoneal membrane as a dialyzing membrane. L. Oliveira and A. Rodrigues report on the impact of previous renal replacement therapy on the membrane function, whereas S. Mizuiri et al. report on the importance of peritoneal effluent markers and their relation to epithelial to mesenchymal transition, which is thought to be an important part of pathogenetic process causing long-term changes in the peritoneum. Finally, N. Jiang et al. report on the impact of a supplemented low-protein diet on peritoneal membrane transport characteristics, whereas A. W. D. Stavenuiter et al. report on the effect of different peritoneal fluid components on the membrane in a rat model.

The guest editors wish to thank all authors for their valuable contributions. Without their efforts, this special issue would not have been possible.

*Hulya Taskapan* *Hulya Taskapan* *Olof Heimburger* *Olof Heimburger* *Cengiz Utas* *Cengiz Utas* *Paul Tam* *Paul Tam*

